# Pathways Regulating Establishment and Maintenance of Cardiac Chamber Identity in Zebrafish

**DOI:** 10.3390/jcdd8020013

**Published:** 2021-01-29

**Authors:** Yao Yao, Amanda N. Marra, Deborah Yelon

**Affiliations:** Division of Biological Sciences, University of California, San Diego, La Jolla, CA 92093, USA; yay132@ucsd.edu (Y.Y.); amarra@ucsd.edu (A.N.M.)

**Keywords:** ventricle, atrium, cardiac chamber formation, FGF, BMP, Nodal, retinoic acid, Nkx2.5, Nr2f2, Nr2f1a

## Abstract

The vertebrate heart is comprised of two types of chambers—ventricles and atria—that have unique morphological and physiological properties. Effective cardiac function depends upon the distinct characteristics of ventricular and atrial cardiomyocytes, raising interest in the genetic pathways that regulate chamber-specific traits. Chamber identity seems to be specified in the early embryo by signals that establish ventricular and atrial progenitor populations and trigger distinct differentiation pathways. Intriguingly, chamber-specific features appear to require active reinforcement, even after myocardial differentiation is underway, suggesting plasticity of chamber identity within the developing heart. Here, we review the utility of the zebrafish as a model organism for studying the mechanisms that establish and maintain cardiac chamber identity. By combining genetic and embryological approaches, work in zebrafish has revealed multiple players with potent influences on chamber fate specification and commitment. Going forward, analysis of cardiomyocyte identity at the single-cell level is likely to yield a high-resolution understanding of the pathways that link the relevant players together, and these insights will have the potential to inform future strategies in cardiac tissue engineering.

## 1. Zebrafish as a Model Organism for Studying the Regulation of Cardiac Chamber Identity

In the early seventeenth century, the prominent physician William Harvey postulated a distinct division of labor between the cardiac chambers [1]. Making an analogy to the firearms of the era, he proposed that atrial contraction is the equivalent of pulling the trigger to release the flint that ignites the gunpowder, whereas ventricular contraction is the explosion that propels the ammunition forward. Indeed, it is now well established that atria and ventricles have specific roles that are facilitated by their distinct morphological, physiological, and molecular attributes. Morphologically, ventricles are larger than atria, with larger individual cardiomyocytes and with thicker and highly trabeculated walls [2,3,4,5]. Physiologically, ventricles and atria exhibit chamber-specific conductive properties, characterized by distinct action potential waveforms and calcium dynamics [6,7,8]; additionally, ventricular cardiomyocytes have an extensive T-tubule system that is not found in atrial cardiomyocytes and is important for ventricular calcium handling [5,9]. On the molecular level, a variety of differentially expressed genes, including ion channels, myosin isoforms and transcription factors, define divergent ventricular and atrial properties [10,11,12,13,14,15]. Since the differences between the chambers are crucial for effective cardiac function, it is important to understand the mechanisms that allocate cells into chamber-specific lineages and direct chamber-specific differentiation.

The zebrafish is an excellent model organism in which to study these fundamental aspects of cardiac chamber formation. The external fertilization, optical transparency, and rapid development of zebrafish embryos facilitate easy access to the developing heart [16]. Importantly, zebrafish can survive until larval stages without a functional cardiovascular system, which is convenient for analysis of mutant embryos with defects in cardiac chamber morphology and function [16]. Classical forward genetic screens, using chemical or insertional mutagens, have yielded a large collection of mutations that disrupt chamber development [17,18,19,20]. Reverse genetic approaches, including morpholino-mediated gene knockdown, TILLING, and targeted genome editing [17,21], have also revealed the functions of a number of relevant genes. In addition, analysis of chamber formation in zebrafish has benefited from the flexibility to manipulate gene expression or pathway activity at different developmental stages. Heat-inducible regulatory elements allow temporally controlled overexpression of transgenes [22], and the permeability of zebrafish embryos enables administration of small molecules to inhibit or activate pathways during a particular timeframe [23].

**Figure 1:**
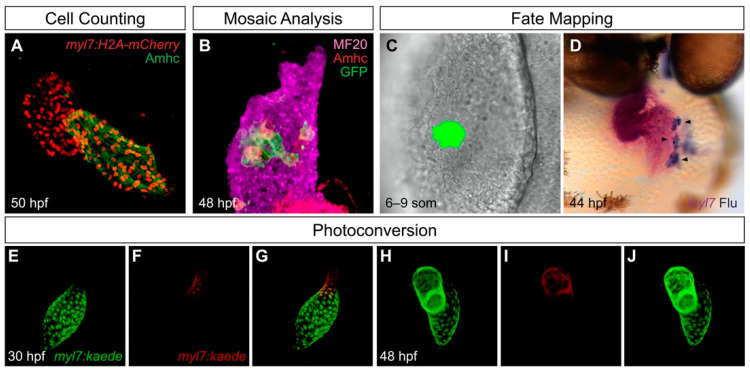
Useful tools for analysis of cardiac chamber development in zebrafish. (**A**) Transgenes such as *Tg(myl7:H2A-mCherry)* label myocardial nuclei (red) and facilitate counting of the cardiomyocytes in each chamber; the atrial myosin heavy chain Amhc (green) distinguishes the atrium from the ventricle. Lateral view of wild-type heart at 50 h post-fertilization (hpf) is adapted from [27]. (**B**) Mosaic analysis enables assessment of the cell-autonomy of gene function. In this example, adapted from [27], mosaic distribution of the transgene *Tg(hsp70:dnfgfr1-eGFP)* (green) inhibits FGF signaling in specific ventricular cells, several of which exhibit ectopic Amhc, indicating a cell-autonomous requirement for FGF signaling to repress *amhc* expression in the ventricle (lateral view). Magenta fluorescence labels sarcomeric myosin heavy chain using the monoclonal antibody MF20, and red fluorescence indicates localization of Amhc, using the monoclonal antibody S46. (**C**,**D**) Fate mapping follows progenitor cells from their origins to their destinations. In this example, adapted from [28], photoactivation of a caged fluorescein-dextran lineage tracer marks a small group of cells in the anterior lateral plate mesoderm (ALPM) at the 6–9 somite (som) stage (**C**, dorsal view). Later, labeled progeny of these cells (arrowheads) are found in the atrium; uncaged fluorescein (blue) is detectable within the *myl7*-expressing myocardium (magenta) of the heart (**D**, frontal view). (**E**–**J**) Photoconvertible proteins facilitate tracking of cells over time. In this example, adapted from [29], cardiomyocytes express the transgene *Tg(myl7:kaede)*, and regionally restricted photoconversion of Kaede at 30 hpf converts its green fluorescence into red fluorescence near the arterial pole of the heart tube (**E**–**G**). Later, visualization of retained red fluorescence demonstrates that the labeled cells contribute to the ventricle (**H**–**J**).

Additionally, a wide selection of embryological tools available in zebrafish provide opportunities for high-resolution analysis of the pathways controlling chamber development (Figure 1). For example, transgenes driving cardiac expression of nuclear-localized fluorescent proteins are useful for precise assessment of the impact of a particular factor on the numbers of ventricular and atrial cells (Figure 1A). Methods for mosaic analysis are helpful for evaluating whether a specific gene or pathway has a cell-autonomous impact on ventricular or atrial traits: mosaic expression of a transgene can be stochastically induced (Figure 1B), or chimeric embryos can be created via blastomere transplantation [24]. Finally, to determine whether particular progenitor cells give rise to ventricular or atrial lineages, it is valuable to track individual cells from their origins to their destinations. Fate mapping approaches in zebrafish can employ photoactivatable lineage tracers [25] (Figure 1C,D) or photoconvertible proteins expressed by transgenes [26] (Figure 1E–J) to follow cells over time and assess their contributions to the cardiac chambers.

Altogether, the combination of genetic and embryological approaches in zebrafish creates valuable opportunities to investigate the mechanisms regulating cardiac chamber identity. Importantly, many of the distinct features of ventricles and atria, including their morphological characteristics, conductive properties, and gene expression profiles, are highly conserved between zebrafish and mammalian hearts [3,4,14,30], suggesting that the key regulatory genes in zebrafish will be broadly relevant across species. Here, we highlight a series of studies in zebrafish that have provided interesting insights into the genetic pathways that distinguish the ventricle from the atrium. Work in zebrafish has identified multiple signaling pathways that act in the early embryo to influence ventricular and atrial specification. Notably, zebrafish studies have also illuminated key factors that act at later stages to reinforce commitment to ventricular and atrial identities, even after chamber-specific differentiation is underway. Finally, we propose future directions that will expand our understanding of the genetic networks controlling chamber identity and could ultimately enhance strategies in tissue engineering.

## 2. Establishment of Ventricular and Atrial Chamber Identity

**Figure 2:**
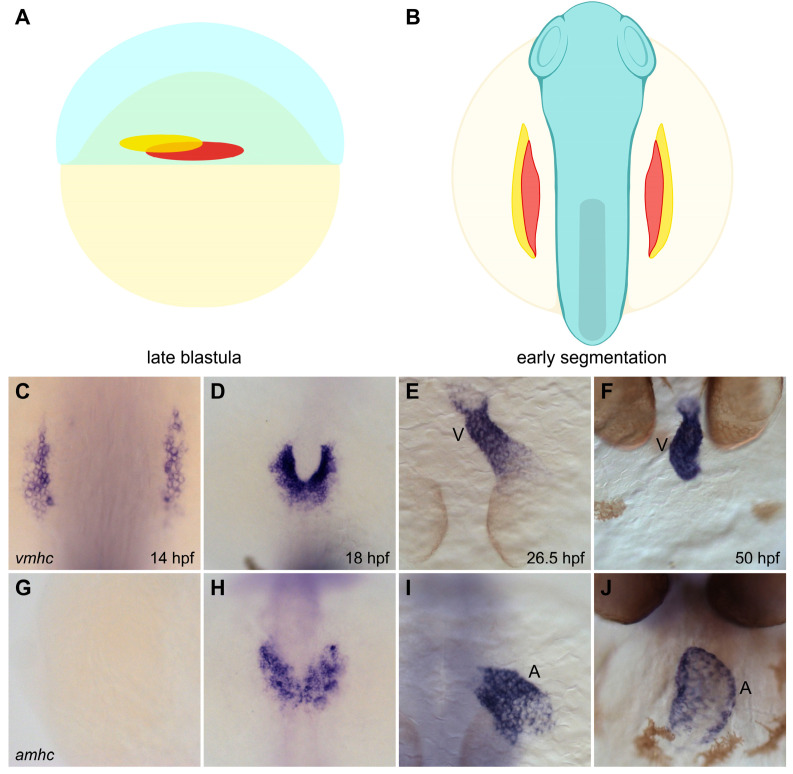
Spatial organization of ventricular and atrial myocardial lineages in the zebrafish embryo. (**A**,**B**) Cartoons illustrate the locations of the territories containing ventricular (red) and atrial (yellow) myocardial progenitor cells in the early embryo. Lateral view of the late blastula (**A**) shows that the regions containing ventricular and atrial myocardial progenitors are spatially organized prior to gastrulation [34]. Dorsal view of the gastrula (**B**) shows the ALPM regions that contain ventricular and atrial myocardial precursors during early segmentation stages [28]. (**C**–**J**) In situ hybridization depicts the expression patterns of *vmhc* (**C**–**E**, dorsal views; **F**, frontal view) and *amhc* (**G**–**I**, dorsal views; **J**, frontal view), indicating the relative positions of ventricular and atrial cardiomyocytes as they differentiate and form the heart. Ventricular cardiomyocytes initiate *vmhc* expression around 14 hpf (**C**), whereas atrial cardiomyocytes initiate *amhc* expression around 18 hpf (**H**) [38,39]; at these stages, ventricular cardiomyocytes are located more medially than atrial cardiomyocytes (**D**,**H**). Ventricular and atrial cardiomyocytes go on to occupy separate portions of the heart tube (**E**,**I**); later, the ventricular and atrial chambers become morphologically distinct (**F**,**J**). V, ventricle, A, atrium. Images adapted from [27]; illustrations by Jessyka T. Diaz.

Across species, the specification of ventricular and atrial progenitor cells is thought to occur in the early embryo during gastrulation stages [31,32,33,34,35,36,37]. Fate mapping studies in zebrafish, chick, and mouse have demonstrated that ventricular and atrial cardiomyocyte lineages are spatially segregated prior to or during gastrulation [31,33,34,35,36,37]. Additionally, gene expression patterns distinguish ventricular and atrial progenitor populations within the early mesoderm, well before the heart tube forms [12,31,33,38,39,40]. In zebrafish, for example, the cardiac fate map shows that ventricular and atrial myocardial lineages are spatially organized in the late blastula, with the ventricular progenitor cells located closer to the margin and to the dorsal midline than the atrial progenitor cells (Figure 2A) [34]. Following gastrulation, ventricular and atrial precursors occupy distinct territories within the anterior lateral plate mesoderm (ALPM), with the ventricular cells positioned more medially than the atrial cells (Figure 2B) [28]. As differentiation proceeds, ventricular and atrial cardiomyocytes in the ALPM can be readily distinguished by their expression of *ventricular myosin heavy chain* (*vmhc*, also known as *myh7*) and *atrial myosin heavy chain* (*amhc*, also known as *myh6*) (Figure 2C–J) [38,39]. Both *vmhc* and *amhc* initiate expression before the heart tube forms (Figure 2C,D,G,H) [38,39], and both are later required for the contractility of their respective chambers [39,41]. After the heart tube assembles, late-differentiating cardiomyocytes join the early-differentiating cardiomyocyte populations, contributing to the arterial and venous poles of the heart, including portions of the ventricle and atrium [42,43,44,45,46,47,48,49]. These late-differentiating myocardial additions also establish the foundations of the outflow tract at the arterial pole and the inflow tract at the venous pole, creating crucial connections between the heart and the vasculature [42,43,44,45,46,47,48,49].

Which genetic pathways initiate ventricular and atrial specification in the early embryo? One intriguing possibility, suggested by the spatial organization of the cardiac fate map [31,33,34,35,36,37], is that differential exposure to secreted signals could influence the assignment of chamber fate. Indeed, studies in zebrafish suggest that the same signaling pathways that influence early mesoderm patterning also contribute to patterning of the cardiac progenitor populations. For example, Nodal ligands, which induce mesendoderm formation at the embryonic margin [50,51], seem to promote ventricular specification in zebrafish [34,52]. Inhibition of Nodal signaling results in reduced formation of ventricular tissue [34,52], and fate mapping experiments have indicated that this phenotype reflects a ventricular-to-atrial fate transformation of progenitor cells located close to the margin [34]. Together, these data suggest that high levels of Nodal signaling, as found in cells near the margin [50,51], encourage ventricular fate assignment.

**Figure 3:**
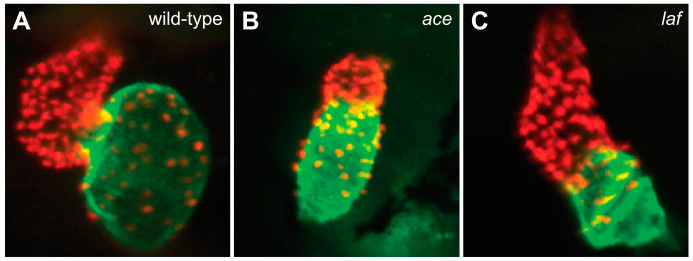
FGF and BMP signaling promote ventricular and atrial cardiomyocyte formation, respectively. Compared to the chambers of the wild-type heart at 48 hpf (**A**), the *ace* (*fgf8a*) mutant heart (**B**) exhibits a substantially reduced ventricle, and the *laf* (*acvr1l*) mutant heart (**C**) exhibits a substantially reduced atrium. Red fluorescence labels cardiomyocyte nuclei, and green fluorescence indicates localization of Amhc. Images adapted from [54,59].

Similarly, FGF signaling, which is distributed in a dorsal-to-ventral gradient in the early mesoderm [53], plays an important role in promoting ventricular specification [54]. In zebrafish *fgf8a* mutants (also called *acerebellar* (*ace*) mutants), the total number of cardiomyocytes is significantly reduced, with a more dramatic reduction in the ventricle than in the atrium (Figure 3B) [54]. This loss of ventricular cells is evident in the ALPM prior to heart tube assembly [54], suggesting an early influence of FGF signaling on ventricular specification, potentially concurrent with its role in the induction of *nkx2.5* expression in cardiac mesoderm [55,56,57,58]. Consistent with this idea, treatment of embryos with the FGFR inhibitor SU5402 during gastrulation caused significant reduction of both ventricular and atrial cell numbers, with a stronger impact on the ventricle [54]. Thus, it seems that high levels of FGF signaling, like high levels of Nodal signaling, act in the early embryo to support establishment of the ventricular progenitor population.

Like FGF signaling, BMP signaling plays early roles in both the induction of cardiac mesoderm [52,60,61] and the assignment of cardiac chamber fates [59]. In contrast to the dorsal-to-ventral distribution of FGF signaling [53], BMP signaling is found in a ventral-to-dorsal gradient in the early embryo [62]. Additionally, in contrast to the *fgf8a* mutant phenotype [54], mutation of the BMP receptor gene *acvr1l* (also known as *lost-a-fin* (*laf*)) results in a substantial reduction of the number of atrial cardiomyocytes (Figure 3C) [59]. This phenotype appears to reflect an early impact of BMP signaling on atrial development: the loss of atrial cells in *acvr1l* mutants is apparent before the heart tube forms, and temporally controlled inhibition of BMP signaling during gastrulation, using either pharmacological or genetic approaches, caused a significant decrease in atrial cardiomyocytes [59]. Conversely, heightened BMP signaling, mediated by overexpression of a constitutively active version of *acvr1l*, caused an increase in the size of the atrium and the number of atrial cardiomyocytes [59]. Collectively, these findings suggest that high levels of BMP signaling play an important role in promoting atrial specification in the early embryo.

Synthesizing these data, it is appealing to consider a model in which the specific levels of Nodal, FGF, and BMP signaling received by a particular myocardial progenitor cell would specify its ventricular or atrial identity. However, it is not yet clear how the integration of these signals might direct cell fate, and it is likely that additional signaling pathways also contribute to the decision between ventricular and atrial lineages. Moreover, the specification of ventricular and atrial identities does not necessarily represent a simple choice between these two options. While the organization of the cardiac fate map (Figure 2A,B) raises interesting questions about the mechanisms that establish the ventricular-atrial pattern within the heart field, it is also important to consider the mechanisms that distinguish the ventricular and atrial progenitors from neighboring progenitor populations in the early mesoderm. For example, retinoic acid (RA) signaling has been demonstrated to play an important role in allocating progenitor cells between ventricular and pharyngeal muscle lineages in zebrafish [63]. RA signaling drives expression of Nr2f transcription factors in the ALPM, where Nr2f1a and Nr2f2 work together to influence cell fate decisions that limit the formation of ventricular cardiomyocytes and favor the formation of pharyngeal muscle cells, indicating another important pathway that controls the dimensions of the ventricular progenitor population [63]. Altogether, although it is clear that multiple signaling pathways influence the establishment of appropriate numbers of ventricular and atrial progenitor cells in the early embryo, there certainly remains much to learn about how these signals interface with each other and which effector genes act downstream in each pathway to sway lineage decisions and initiate chamber-specific differentiation programs.

## 3. Maintenance of Ventricular and Atrial Chamber Characteristics

How do ventricular and atrial fate assignment lead to the acquisition of distinct ventricular and atrial features? Presumably, the signals that control specification also influence the activation of chamber-specific transcriptional programs that create the morphological and physiological differences between ventricular and atrial cardiomyocytes. Although the precise details of the pathways that link chamber specification and differentiation are not fully understood, studies in chick and mouse have yielded clear examples of potent chamber-specific transcription factors that regulate chamber-specific characteristics. For example, the orphan nuclear receptor COUP-TFII (also known as Nr2f2) is found in atrial, but not ventricular, cardiomyocytes, where it drives expression of atrial genes and suppresses expression of ventricular genes [5,64]. Many of these differentially expressed genes are direct targets of COUP-TFII, including the myosin isoforms *Mlc1a*, *Mlc2a,* and *Mlc2v* and the transcription factors *Hey2* and *Irx4* [5]. Conversely, Irx4, a member of the Iroquois transcription factor family, is ventricle-specific and promotes ventricular gene expression while repressing atrial gene expression [65,66,67,68], potentially via direct repression of atrial genes in the ventricular myocardium [69]. These studies point to a model in which ventricular and atrial differentiation are driven by transcriptional networks that simultaneously promote one chamber-specific pathway and repress the other.

Intriguingly, the initiation of chamber-specific differentiation is not necessarily sufficient to insure cellular commitment to an atrial or ventricular identity: even cells that appear to be terminally differentiated can remain quite plastic and require active maintenance in order to retain their distinct characteristics. Under certain circumstances, atrial or ventricular cardiomyocytes can appear to transform, losing their chamber-specific traits while simultaneously acquiring features of the other chamber. In mice, for example, conditional knockout of *COUP-TFII* in differentiated cardiomyocytes caused the atria to acquire ventricular properties [5]. Expression of atrial markers, such as *Mlc1a* and *Mlc2a*, was lost, and atrial cells began expressing ventricular markers, such as *Mlc1v* and *Mlc2v* [5]. Moreover, the atrial cardiomyocytes in *COUP-TFII*-deficient hearts began to exhibit morphological and physiological characteristics of ventricular cardiomyocytes, including larger cell size, organized T tubules, and ventricle-like action potentials [5]. Thus, COUP-TFII plays an important role in reinforcing commitment to atrial cardiomyocyte identity. In the ventricle, work in chick and mouse has shown that the transcription factors Irx4 and Hey2 contribute to the maintenance of ventricular identity by suppressing ectopic activation of atrial genes in ventricular cardiomyocytes [65,66,70,71]. Together, these findings demonstrate the malleability and reversibility of chamber fate decisions, even after differentiation initiates.

**Figure 4:**
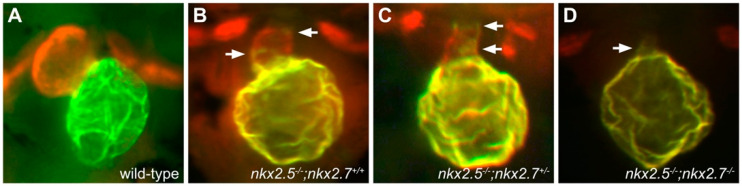
*nkx* genes are required for maintenance of ventricular cardiomyocyte identity. Compared to the wild-type heart at 52 hpf (**A**), *nkx2.5* mutants (**B**) exhibit a reduced ventricle and an enlarged atrium, with ectopic Amhc in some ventricular cells (arrows), and additional loss of a single allele of *nkx2.7* (**C**) leads to increased presence of ectopic Amhc in the ventricle (arrows). *nkx2.5;nkx2.7* double homozygotes (**D**) exhibit only a small ventricular remnant, with Amhc present throughout (arrow). Red fluorescence labels sarcomeric myosin heavy chain, and green fluorescence indicates localization of Amhc. Images adapted from [29].

Studies in zebrafish have provided further evidence for the importance of active reinforcement of chamber-specific characteristics and have identified additional factors that contribute to chamber identity maintenance. For example, analyses of zebrafish *nkx2.5* and *nkx2.7* mutants have demonstrated that Nkx transcription factors play a key part in enforcing ventricular cardiomyocyte identity [29,72,73]. Strikingly, *nkx2.5* mutants exhibit a diminished ventricle and an expanded atrium, as well as ectopic expression of *amhc* in some ventricular cells (Figure 4B) [29]. These phenotypes are exacerbated by mutation of *nkx2.7*, with *nkx2.5*; *nkx2.7* double mutants displaying only a small, *amhc*-expressing ventricular remnant (Figure 4C,D) [29]. Despite these evident defects in the cardiac chambers, the numbers and characteristics of the ventricular and atrial cardiomyocytes initially appear normal within the *nkx*-deficient heart tube; the loss of ventricular cells and corresponding gain of atrial cells, as well as the reduction of ventricular gene expression and appearance of ectopic atrial gene expression, all emerge gradually over time [29,72]. These phenotypes suggest that ventricular cardiomyocytes can transform into atrial cardiomyocytes in the absence of *nkx* gene function. Indeed, cardiomyocytes labeled via photoconversion in the ventricular portion of the heart tube were later found within the *amhc*-expressing atrial chamber of the *nkx*-deficient heart, demonstrating important roles of *nkx* genes in maintaining ventricular identity and repressing atrial identity within the ventricular myocardium [29]. These findings resonate with gene expression data from mouse *Nkx2-5* mutant hearts, which display not only reduced expression of ventricle-enriched genes, such as *Mlc2v* and *Hand1*, but also increased expression of atrial genes, including *COUP-TFII* [13,74,75,76]. Additionally, *Nkx2-5* homologs promote ventricular expression of *Irx4* and *Hey2* homologs in both zebrafish and mammalian models [29,67,77], suggesting a conserved role for *Nkx* genes near the top of a transcriptional hierarchy that insures maintenance of ventricular identity.

**Figure 5:**
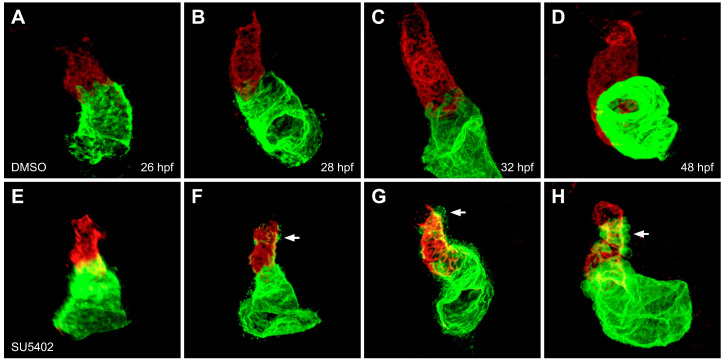
FGF signaling is required to maintain ventricular cardiomyocyte identity. In contrast to control embryos treated with DMSO (**A**–**D**), embryos treated with SU5402 beginning at 18 hpf (**E**–**H**) exhibit gradual accumulation of ectopic Amhc (arrows), accompanied by diminishing levels of Vmhc in the ventricle, between 28 and 48 hpf (**F**–**H**). Red fluorescence indicates localization of Vmhc, and green fluorescence indicates localization of Amhc. Images adapted from [27].

Do the signaling pathways that influence ventricular fate assignment also interface with the Nkx-driven transcriptional network to sustain ventricular cardiomyocyte identity? Interestingly, the early role of FGF signaling in promoting ventricular specification is echoed in its later role in enforcing ventricular characteristics [27]. Temporally controlled inhibition of the FGF pathway in zebrafish, via treatment with SU5402 or induction of a transgene expressing a dominant negative form of FGFR1, has served as a valuable tool for analyzing the roles played by FGF signaling after myocardial differentiation is underway [27,42,54]. Notably, inhibition of FGF signaling at 18 h post-fertilization (hpf), after ventricular and atrial cardiomyocytes already exhibit differential patterns of gene expression (Figure 1D,H), resulted in the gradual appearance of *amhc* expression in the ventricle (Figure 5) [27]. In addition to gaining ectopic expression of *amhc*, ventricular cardiomyocytes exhibited reduced expression of *vmhc*, and ventricular cell number decreased while atrial cell number increased [27]. Additionally, mosaic analysis indicated that inhibition of FGF signaling could act in a cell-autonomous fashion to induce ectopic *amhc* expression in ventricular cardiomyocytes (Figure 1B) [27]. Overall, inhibition of FGF signaling at 18 hpf appeared to transform ventricular cardiomyocytes into cells with atrial characteristics, reminiscent of the *nkx*-deficient phenotype [27,29]. Fittingly, *nkx* gene expression was diminished when FGF signaling was inhibited, and overexpression of *nkx2.5* in SU5402-treated embryos reduced the appearance of ectopic *amhc* in the ventricle [27]. Thus, in addition to the early function of the FGF pathway in promoting ventricular progenitor specification [54], FGF signaling also plays an important role, upstream of *nkx* genes, in insuring maintenance of ventricular cardiomyocyte identity.

It is not yet known how directly FGF signaling influences *nkx* gene expression in this context or which other effector genes lie downstream of FGF signaling to reinforce ventricular characteristics. There may also be additional signaling pathways that contribute to the maintenance of the ventricular myocardium. In this regard, it is interesting to note that activation of the BMP signaling pathway at 18 hpf, via a heat-inducible transgene expressing *bmp2b*, resulted in a reduced number of ventricular cardiomyocytes [78]. In addition, ectopic *amhc*-expressing cells appeared within the ventricle when induction of *bmp2b* expression was combined with knockdown of *smad6a*, an inhibitor of BMP signal transduction [78]. These results suggest that inhibition of BMP signaling could play an important part in maintaining the chamber-specific features of ventricular cardiomyocytes. It will be valuable for future studies to delve deeper into this possibility and to examine the relationship between the BMP and FGF signaling pathways during ventricular chamber maintenance.

In addition to the reinforcement of ventricular traits by the FGF-Nkx pathway, a separate set of players act to maintain chamber-specific characteristics in the zebrafish atrium. Reminiscent of the role of *COUP-TFII/Nr2f2* in mouse [5], the zebrafish paralog *nr2f1a* is expressed in atrial cardiomyocytes and promotes maintenance of atrial identity [79]. In *nr2f1a* mutants, the initial formation of atrial cardiomyocytes appears unaffected, but the atrium later becomes abnormally small [79]. Intriguingly, the expression of *amhc* retracts from the atrioventricular boundary of the *nr2f1a* mutant heart, and the expression of *vmhc* expands into the atrium [79]. This encroachment of *vmhc* expression is accompanied by the expanded expression of a number of markers for the atrioventricular canal (AVC), including *bmp4*, *tbx2b*, and *notch1b* [79]. These data suggest that *nr2f1a* acts both to promote atrial characteristics and suppress AVC characteristics, thereby enforcing the maintenance of a distinct boundary between the atrial and AVC territories. Thus, atrial and ventricular chamber identities both require active maintenance over time and utilize separate pathways for these processes; further studies delving deeper into the details of chamber-specific maintenance will be important to uncover the mechanisms that simultaneously support and repress distinct identities in each case.

## 4. Future Directions toward a High-Resolution Understanding of the Regulation of Chamber Identity

Studies in zebrafish have identified a number of important factors that contribute to the establishment and maintenance of cardiac chamber identity, yet it is clear that many open questions remain. Future work is needed to illuminate the precise pathways that lead from specification signals to ventricular and atrial fate assignments, as well as the mechanisms that translate these lineage decisions into the execution of distinct differentiation programs. Deeper assessment of these pathways will also reveal the key similarities and differences between the processes that establish and maintain chamber identity. Are the same effector genes that initiate chamber fate decisions also involved in enforcing commitment to chamber identity, or is maintenance fundamentally different from specification? Related to this, what determines the timeframe during which chamber identity must be actively maintained? Studies of *COUP-TFII*, *nkx2.5*, and FGF signaling have suggested that each of these players reinforces chamber identity commitment only during a particular interval of time, after which chamber identity seems to be less malleable [5,27,73]. What, on a molecular level, changes as a more plastic cardiomyocyte develops into a more committed cardiomyocyte, and how do chamber maintenance pathways influence this transition? Could plasticity be induced again at later stages? Intriguingly, a study in zebrafish larvae has suggested that injury can stimulate plasticity of chamber identity: ablation of ventricular cardiomyocytes triggered the migration of *amhc*-expressing cells into the ventricle, where they appeared to transdifferentiate and contribute to ventricular regeneration [80]. Going forward, further utilization of the many tools available in zebrafish will surely provide new insights into the mechanisms regulating chamber fate decisions and the balance between plasticity and commitment.

In addition, it will be important for future work to expand upon our current definitions of ventricular and atrial identity. Clearly, categorizing cardiomyocytes as ventricular or atrial is overly simplistic, as it is evident that there are multiple types of cells within each chamber. For example, across vertebrate species, cardiomyocytes in the outer curvature and inner curvature of the ventricle exhibit different gene expression patterns and distinct conductive properties [2,7,41,81,82,83]. Additionally, in all vertebrate species, ventricles and atria are composed of cardiomyocytes derived from both early-differentiating and late-differentiating myocardial progenitor populations [84,85]. Of course, in higher vertebrates, there are also clear distinctions between the characteristics of the right and left ventricles and atria [86], some of which are echoed by distinct territories within the zebrafish heart [87,88]. Ultimately, it will be interesting to determine how each subset of ventricular and atrial cardiomyocytes is established and to what extent these subpopulations share common pathways for specification and maintenance. Multiple lineages may share similar requirements: for instance, both early-differentiating ventricular cardiomyocytes and late-differentiating outflow tract cardiomyocytes require FGF signaling for their specification [42,44,49,54,89], and both early-differentiating and late-differentiating ventricular cardiomyocytes depend upon *nkx* genes and FGF signaling to reinforce their ventricular characteristics [27,73]. On the other hand, there seem to be regional differences in ventricular plasticity: ectopic *amhc* expression is more frequently observed in the inner curvature than in the outer curvature following inhibition of FGF signaling, suggesting different regional requirements for maintenance of ventricular identity [27]. The advent of single-cell RNA-sequencing techniques has greatly enhanced our understanding of the variety of types of ventricular and atrial cardiomyocytes [11,13,90]; utilization of these strategies in zebrafish will undoubtedly open new avenues toward revealing the requirements for establishing and maintaining the distinct features of each population.

For each mechanism shown to regulate chamber identity in zebrafish, it will be valuable for future studies to assess the conservation of its role across vertebrate species, as this will influence its translational potential. Mutations in several of the transcription factor genes discussed above—*NKX2-5*, *IRX4*, *HEY2*, and *COUP-TFII*—have been implicated in causing certain types of congenital heart disease (CHD), which can include defects in the size, shape, or structure of the cardiac chambers [91,92,93,94,95,96,97]. Notably, mutations in *NKX2-5* have been found in some cases of Ebstein’s anomaly, a CHD that features a partially atrialized right ventricle [98,99,100]. A deeper understanding of the effector genes acting downstream of these transcription factors may shed light on how errors in the regulation of chamber identity could contribute to the defects observed in CHD patients. In addition, insights into the pathways controlling cardiac chamber identity may facilitate future efforts in cardiac tissue engineering. Engineered heart tissue generated from human pluripotent stem cells (hPSCs) is a powerful context for disease modeling as well as a promising regenerative medicine strategy [101,102,103,104]. For these purposes, it is vital to be able to produce pure populations of distinct subtypes of cardiomyocytes [103,105,106,107,108]. The signals that influence chamber identity in the embryo—including Nodal, BMP, FGF, and RA—are likely to be relevant to the acquisition of chamber identities during hPSC differentiation *in vitro* [103]. For example, studies in hPSCs have demonstrated that the ratio of Nodal signaling to BMP signaling impacts the decision between ventricular and atrial fates, with high levels of Nodal signaling and low levels of BMP signaling promoting ventricular specification [105], consistent with the roles of the Nodal and BMP pathways in the zebrafish embryo [34,52,59]. Therefore, further analysis of the pathways that guide cardiac chamber identity acquisition and maintenance in zebrafish has the potential to yield valuable inspiration for enhanced protocols aimed at creating stably differentiated populations of ventricular and atrial cardiomyocytes for translational or therapeutic purposes. Over the long term, a high-resolution understanding of the regulation of cardiac chamber identity will enrich our comprehension of possible etiologies of CHD and future approaches in tissue engineering, and ongoing studies in zebrafish will provide meaningful contributions toward these goals.

## Data Availability

Not applicable.
